# p63 expression in granulosa-luteinized cells of infertile patients with peritoneal endometriosis submitted to *in vitro* fertilization

**DOI:** 10.5935/1518-0557.20210090

**Published:** 2022

**Authors:** Joelmir José Chiesa, Silvia Liliana Cossio, Emily De Conto, Vanessa K. Genro, João Sabino Cunha-Filho

**Affiliations:** 1 UFRGS, Universidade Federal do Rio Grande do Sul, Hospital de Clínicas de Porto Alegre, Porto Alegre, Brazil; 2 INSEMINE, Human Reproductive Center, Porto Alegre, Brazil

**Keywords:** endometriosis, granulosa cells, infertility, *in vitro* fertilization, p63 gene

## Abstract

**Objective:**

Endometriosis is associated with infertility, even without an anatomical abnormality. Furthermore, the peritoneal (mild) phenotype of this disease is the most prevalent and linked to infertility. The present study aimed to investigate the p63 gene and protein expression in granulosa cells from pre-ovulatory follicles in patients with endometriosis and infertility submitted to *in vitro* fertilization.

**Methods:**

Twenty-eight patients participated in the study and were divided into two groups according to the presence or absence of endometriosis. The p63 gene-expression levels assessment was performed by real-time PCR (qPCR) using the TaqMan assay, and we used immunofluorescence to check the p63 protein expression after IVF.

**Results:**

There was no significant difference between the groups regarding age, hormonal levels, oocyte standards, and p63 gene expression. The control group showed an RQ of 1.000 (0.431 to 2.323) and the study group showed an RQ of 0.725 (0.249 to 2.105), *p*>0.05. Both groups showed a weak expression of the p63 gene (*p*>0.05).

**Conclusions:**

This study described that endometriosis may not affect the p63 gene expression. Moreover, after follicular recruitment and growth, we found a weak expression of this protein, suggesting it is not part of oocyte maturation and development control.

## INTRODUCTION

Human cells are continuously exposed to external and internal stressor agents, which can cause damage to the cells' integrity and genomes. The p53 family is the most representative family of genes involved in survival mechanisms (Yang *et al*., 2002). This family of proteins comprises the p53, the p63, and the p73, which are also transcription factors; they regulate the transcription of a subset of common proteins, as well as unique proteins (Melino *et al*., 2003; Hu *et al*., 2011). The *human tumor protein 63* (TP63) gene encodes the p63 protein (Yang *et al*., 1999), and is considered responsible for the maintaining the genetic integrity of germ cells. Also, the p63's role was evidenced by targeting the CK2β deletion in mice oocytes, causing female infertility, which was attributed to premature ovarian failure (Liang *et al*., 2018).

In humans, there are reports of infertile patients with differences in p63-gene-expression (Holder-Espinasse *et al*., 2007; Feng *et al*., 2011). The presence of single nucleotide polymorphisms in the p63 suggests that these proteins may play another role in women's fertility and not just in maintaining the integrity of female germ cells lineage.

Endometriosis is an estrogen hormonal-related disease, and it is associated with infertility, mainly the peritoneal phenotype. Several mechanisms are related to endometriosis and ovulatory or granulosa cell dysfunction (De Conto *et al*., 2017). However, the body of evidence linking peritoneal endometriosis to granulosa cell/oocyte competence is flawed and lacks scientific strength. Furthermore, the p63 protein was not studied in granulosa cells of patients with endometriosis (Brandão *et al*., 2018).

Considering that p63 plays an important role in women's germ cell lineage and has already been described for its abnormal expression in patients with endometriosis, we could also hypothesize that the p63 could be expressed in luteinized granulosa cells. Therefore, this study aimed to evaluate the p63's gene and protein expression in infertile patients with and without endometriosis.

## MATERIALS AND METHODS

### Design

The study was a cross-sectional study.

### Selection of patients

Eligibility criteria for patient selection were women undergoing their first treatment for infertility with clinical indication of in vitro fertilization (IVF), age equal to or less than 40 years, normal ovarian reserve, regular menstrual cycles, varying between 21-35 days, peritoneal endometriosis or tubal factor. The excluding criteria were patients older than 40 years old; patients with ovarian cysts, including endometriomas, blood after granulosa cell centrifugation; and any endocrine disorders such as polycystic ovary syndrome, hyperprolactinemia, and hypothyroidism.

All patients were informed about the procedures and signed an informed consent form. The bioethics committee of the Hospital de Clínicas de Porto Alegre (IRB, CAAE: 2098312.0.0000.5327) approved this research project and we followed the STROBE guideline (von Elm *et al*., 2014).

Thirty-one patients were analyzed and included in the study, from 2014 to 2018. Two patients were excluded for having an endometrioma and one for having blood after the preparation of the granulosa cells. They were divided into two groups, according to the cause of infertility. Patients with minimal or mild endometriosis (peritoneal) were included in the study group. The control group comprised patients with infertility by a tubal factor without endometriosis. The presence or absence of endometriosis was confirmed by laparoscopy and biopsy, performed in the 6 months prior to the IVF.

Ovulation induction was performed with the antagonist protocol, with recombinant follicle-stimulating hormone (FSH). The human chorionic gonadotropin (hCG) administration criteria included the presence of at least 3 follicles at 17 mm. After follicular aspiration and oocyte separation, granulosa cells from each patient were prepared for analysis of gene and protein expression. The pool of granulosa cells (from follicles with more than 17 mm) was placed on plates with G-MOPS™ buffered medium, supplemented with human serum albumin and gentamycin. The granulosa cells were washed six times in 100 µL droplets G-MOPS™ medium to enable the elimination of any impurities such as blood clots. After washing, the cells were centrifuged at 200 x g for 10 minutes and re-suspended in fresh G-MOPS™. All procedures were performed during a heating stage at 37°C, and the G-MOPS medium was prepared at least 12 hours before the procedure.

### Gene expression assessment

The extraction of total RNA from the aspirated granulosa cells was performed using the RNeasy™ extraction kit (Qiagen), following the manufacturer's instructions. The gene expression levels of the p63 gene assessment was performed by real-time PCR (qPCR), using the TaqMan assay, following the 2^-ΔΔCT^ method (Livak & Schmittgen, 2001). We used the StepOne™ System (Life Technologies) for the qPCR assessment. The experiment was performed in duplicate, and the gene utilized to calculate the delta was the β-actin.

### Protein Expression Assessment

We used the immunofluorescence technique, according to the protocol, as previously described by Hill (1984) and Gonfloni *et al*. (2009). We used an Olympus BX51 microscope with 40x magnification, to analyze the p63 expression. To have control over the experiments, we used fibroblasts as positive controls to this specific antibody, and DAPI as a control to immunofluorescence.

### Statistical analysis

We analyzed the categorical variables using the Chi-square test. For continuous variables, we used the Student's t-test and the Mann-Whitney test for parametric and non-parametric distribution, respectively. We searched for correlations between the variables using the Pearson correlation coefficient. The significance level was 5%, and the study power was 80%.

We analyzed the gene expression data with the 2^-ΔΔCT^ method (Livak & Schmittgen, 2001), which is efficient for analyzing changes in expression of a particular gene when the quantitative data are from qPCR and there is a comparison of a specific gene expression between two groups. We ran multiple comparisons using the logistic regression analysis.

### Sample Size and Power Calculation

We calculated the sample based on the article from Poli Neto *et al.* (2007), that detected the p63 protein expression in patients with endometriosis and in controls. Sixteen patients were required to have an 80% chance of detecting, as significant at the 5% level, an increase in the primary outcome measure (Poli Neto *et al*. 2007; Lemos *et al*., 2008).

Moreover, using our results we analyzed our data to certify the robustness of our results, the power calculation was > 80%, considering the p63 expression protein as our primary outcome to find a difference of 0.5 in 80%, sure that the lower limit of a one-sided 95% confidence interval (or equivalently a 90% two-sided confidence interval), in a non-inferiority trial model.

## RESULTS

The overall mean age was 32.9 years (range 21 to 40, SD 0.83), and FSH serum levels had a mean level of 6.8 mIU/mL (range 3.1 to 10.8, SD 1.97). Estradiol levels had a mean level of 68.82 pg/mL (range 27 to 220, SD 48.15), and the AMH serum levels had a mean level of 3.52 ng/mL (range 0.6 to 8.11, SD 2.41). The mean number of follicles measuring more than 17 mm was 5.44 (range 1 to 15, SD 3.3). Besides, the number of recovered oocytes showed a mean of 7.46 (range 1 to 15, SD 3.54), and the mean number of generated embryos was 4.54, range 1 to 10, SD 2.64. The embryo's score for the day 3 mean was 63.21, (range 0 to 100, SD 25.35) in all patients. Comparisons were not significant between non-endometriosis and endometriosis patients (Table 1). Only one member of the control group had been previously pregnant. All other participants had primary infertility. The correlations between gene expression by qPCR with age (r=-0.271), AMH serum levels (r=-0.073), and the number of aspirated oocytes (r=-0.028) were evaluated through a multivariable analysis. There were no significant correlations in these cases (*p*>0.05).

**Table 1.** Patients characteristics by groups of the study.

Variables	Groups		*p* value*
	Control (n =19)	Study (n=9)	
Age (years)	32.21±4.47	34.44±2.30	0.17
FSH levels (mIU/mL)	6.72±2.14	7.17±1.13	0.70
Oestradiol levels (pg/mL)	61.00[45.83-104.57]	43.20[12.68-65.45]	0.20
AMH levels (ng/mL)	3.82±2.49	2.86±2.25	0.36
Number of follicles ≥17 mm	5.89±3.66	4.56±2.40	0.33
Number of recuperated oocytes	7.74±3.31	6.89±4.14	0.56
Number of generated embryos	4.89±2.62	3.78±2.68	0.31
Embryos score in day 3 (%)	60.21±26.19	69.58±23.63	0.37

Legend: Characteristic of the patients of this study comparing control and study groups. The variables described as mean ± standard deviation were evaluated through Student's t test, the variable described as median[confidence interval of 95%] was evaluated through Mann-Whitney test. FSH levels - Follicle Stimulating Hormone Levels; AMH levels - Anti-Müllerian Hormone Levels;

* p value according to statistical analysis.

### Gene expression (qPCR)

**Figure 1:**
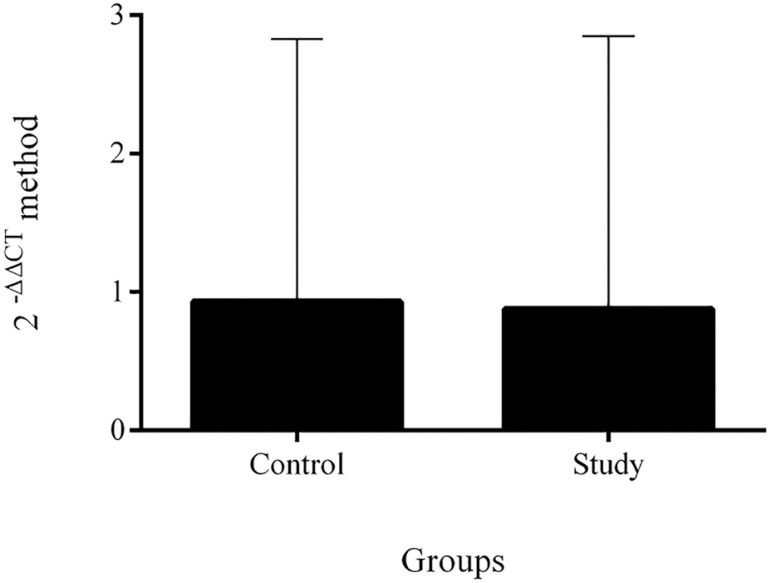
p63 gene expression in between the groups. Legend: Comparison of p63 gene expression between the control and study groups showing no statistical difference between them (*p*>0.05); qPCR results were evaluated by the 2-ΔΔCT method and the results are shown as relative quantification (RQ) in the Y-axis; The control group showed an RQ of 1.000 and 0.431 and 2.323 minimum and maximum RQ, respectively; The study group showed an RQ of 0.725 and 0.249 and 2.105 minimum and maximum RQ, respectively.

The median was 0.93 in the control group (95%CI, 0.55-2.83) and 0.88 in the study group (95%CI 0.24-2.85) (Figure 1). The results also demonstrated that the p63 gene is weakly expressed in the granulosa cells in the two groups.

### Protein expression (immunofluorescence)

**Figure 2:**
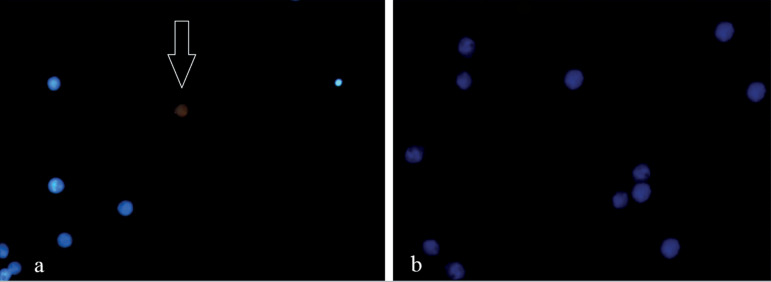
Immunofluorescence result comparing both groups. Legend: Immunofluorescence showing a single cell stained positive for the p63 protein. (a) Immunofluorescence showing a single cell stained positive for the p63 protein granulosa cell in the study group, using the Donkey Anti-Mouse IgG H & L (Alexa Fluor 647, Abcam, cat # ab150107) as a secondary antibody that stains red, signed with (↓). (b) Control group image with a negative result.

The two groups were evaluated by immunofluorescence to show the p63 protein; there was an extremely weak expression (Figure 2).

## DISCUSSION

Several studies have shown that the p63-transcription factor may be the main regulator of the cell cycle in germ cells, by controlling cell division; the p63 may also be involved in apoptosis induction when there is damage to the genetic material. Our study found a weak expression of the p63 gene and protein in granulosa-luteinized cells of infertile patients undergoing IVF, and there was no difference between the Endometriosis and the Control groups. The protein expression could be due to the sample origin, since granulosa cells were obtained from mature follicles, and the TAp63α isoform is the most important regulator of fidelity genic protection in germ cells during meiotic arrest (Becker *et al*., 2014). Therefore, a hypothetical increase of the p63 expression in immature ovary follicles could even be a facilitator or promoter of follicular atresia by cellular apoptosis. The patients' ages could be another explanation for a weak p63-expression and the lack of difference between the groups. All patients were younger than 40 years; we expected less DNA damage to the cells. Additionally, women over 40 years of age had signs of lower ovarian reserves, an increase in ovarian atresia and a higher number of genetic errors and difficulty in preserving DNA integrity could present as an overexpression of the p63 protein.

Since the p63 gene was weakly expressed during follicular growth, we hypothesized that this gene is not associated with follicular development in the already dependent stages of FSH stimulation. Most likely is that the p63 has its main role in the control of the primary follicles that are at rest in the ovary (FSH-independent). If any genetic damage would be present in the follicular cells at an early stage, these cells would likely suffer apoptosis, and the follicle would begin the atresia process. This was well-documented in an animal model inducing premature ovarian failure by blocking CK2β in mice oocytes from the primordial follicle stage (Liang *et al*., 2018).

This is the first time that the p63 gene and protein expression were investigated in granulosa-luteinized cells in patients with endometriosis. A study by Poli Neto *et al*. (2007) showed that there was a difference in the p63 protein expression between different kinds of endometriosis injuries, such as overexpression in peritoneal disease and endometrioma, but without considering the presence of infertility. Our group of patients had only minimal or mild disease. Because endometriosis is a very heterogeneous disease, it is possible that our results could not be replicated if the p63 expression is investigated in patients in other stages of the disease.

This paper has a few biases regarding the homogeneity of subjects, because all patients were Caucasian. Furthermore, all ovarian stimulation was controlled and used the same protocol. Finally, the selected patients had a good prognosis before IVF, with a normal ovarian reserve, and without endocrine diseases. The sample size was adequate to discriminate significant differences between both groups regarding the p63 protein expression. However, for other reproductive outcomes (implantation or pregnancy rates) our sample size was insufficient.

In conclusion, peritoneal endometriosis associated-infertility was not linked to an altered p63 expression in a granulosa-luteinized cell. Moreover, the p63 was weakly expressed at the final stage of follicular development (FSH-dependent). Thus, p63 may not play an important role in the more advanced stages of follicular growth.
